# Pea Albumin Extracted from Pea (*Pisum sativum* L.) Seeds Ameliorates High-Fat-Diet-Induced Non-Alcoholic Fatty Liver Disease by Regulating Lipogenesis and Lipolysis Pathways

**DOI:** 10.3390/nu16142232

**Published:** 2024-07-11

**Authors:** Shucheng Zhang, Zhengwu Cui, Hao Zhang, Pengjie Wang, Fuqing Wang, Jian Zhang

**Affiliations:** 1 College of Food Science and Nutritional Engineering, China Agricultural University, Beijing 100083, China; zhangshucheng120@sina.com (S.Z.); zhanghaocau@cau.edu.cn (H.Z.); 2 Department of Nutrition and Health, China Agricultural University, Beijing 100193, China; 16653221635@163.com; 3 Tibet Tianhong Science and Technology Co., Ltd., Lhasa 850000, China; fq7963@163.com

**Keywords:** non-alcoholic fatty liver disease, pea albumin, high-fat diet, hepatic steatosis, lipogenesis, lipolysis

## Abstract

Non-alcoholic fatty liver disease (NAFLD) is now recognized as the most prevalent liver disease globally. Pea albumin (PA) has demonstrated positive impacts on reducing obesity and improving glucose metabolism. In this research, a mouse model of NAFLD induced by a high-fat diet (HFD) was employed to examine the impact of PA on NAFLD and explore its potential mechanisms. The findings revealed that mice subjected to a HFD developed pronounced fatty liver alterations. The intervention with PA significantly lowered serum TC by 26.81%, TG by 43.55%, and LDL-C by 57.79%. It also elevated HDL-C levels by 1.2 fold and reduced serum ALT by 37.94% and AST by 31.21% in mice fed a HFD. These changes contributed to the reduction in hepatic steatosis and lipid accumulation. Additionally, PA improved insulin resistance and inhibited hepatic oxidative stress and inflammatory responses. Mechanistic studies revealed that PA alleviated lipid accumulation in HFD-induced NAFLD by activating the phosphorylation of AMPKα and ACC, inhibiting the expression of SREBF1 and FASN to reduce hepatic lipogenesis, and increasing the expression of ATGL, PPARα, and PPARγ to promote lipolysis and fatty acid oxidation. These results indicate that PA could serve as a dietary supplement for alleviating NAFLD, offering a theoretical foundation for the rational intake of PA in NAFLD intervention.

## 1. Introduction

Non-alcoholic fatty liver disease (NAFLD) is defined by steatosis occurring in over 5% of hepatocytes in the absence of significant alcohol consumption or other chronic liver diseases, ultimately leading to excessive lipid deposition [1]. The health risks associated with NAFLD extend beyond simple steatosis; it can progress from simple fat accumulation to steatohepatitis, fibrosis, cirrhosis, and even hepatocellular carcinoma [2]. NAFLD is a major liver disease worldwide, comparable to obesity and diabetes, with an estimated global adult prevalence of approximately 29.62% [3]. However, the pathogenesis of NAFLD is not yet widely agreed upon, involving multiple factors such as excessive dietary fat intake, impaired insulin signaling, disrupted hepatic lipid metabolism, and increased oxidative stress or inflammatory responses in hepatocytes [4,5]. Among the many pathogenesis mechanisms, the “two-hit” hypothesis is currently the most widely accepted. According to this hypothesis, lipid accumulation in the liver, insulin resistance, and oxidative stress are the primary factors leading to NAFLD [4]. Long-term intake of high sugar and high fat, along with insulin resistance, act as the “first hit”, causing lipid accumulation in the liver and the formation of NAFLD. This increases the susceptibility to the “second hit” (including oxidative stress and inflammation), leading to liver damage, which can result in more severe conditions such as non-alcoholic steatohepatitis (NASH) and even fibrosis [6]. Given the complex etiology of NAFLD, recommended interventions mainly include surgical, pharmacological, and lifestyle interventions. Surgical treatment can improve hepatic steatosis, but carries the risk of exacerbating liver fibrosis and the potential for postoperative relapse [7]. Currently, no specific drugs have been approved for the treatment of NAFLD. Although some medications can be used for patients with NAFLD and related conditions, their safety remains controversial. Pioglitazone is a widely studied drug for treating NAFLD, but it has side effects such as weight gain and other risks, including a potential increase in the incidence of bladder cancer and heart failure [8,9]. GLP-1 receptor agonists, such as liraglutide and semaglutide, have been shown to treat NAFLD; however, they can cause gastrointestinal side effects, including nausea and vomiting [10,11]. Despite the ongoing development of various therapeutic drugs and targets, lifestyle changes, including diet and exercise, continue to be the main interventions. Research has found that dietary intake of natural products can alleviate NAFLD with relatively high safety for humans.

Based on the characteristics of high efficacy and low toxicity, numerous studies have found that plant bioactive substances can intervene in NAFLD. Currently, the reported natural products that regulate hepatic lipid metabolism mainly include alkaloids, phenolic compounds, proteins, vitamins, and other bioactive substances. These substances primarily regulate lipid metabolism by suppressing hepatic lipid synthesis and enhancing lipid oxidation and decomposition, ultimately improving NAFLD. Betaine can reduce fatty acid synthesis by regulating SREBF1 and FASN and promote fatty acid oxidation by regulating PPARα and CPT-1, thereby reducing lipid accumulation in the liver [12]. Curcumin also affects hepatic lipid synthesis pathways by inhibiting the expression of SREBF1 and FASN, thus reducing hepatic fat synthesis [13]. β-Conglycinin can modulate hepatic lipid homeostasis by downregulating SREBF1 and upregulating PPARα protein expression [14]. Although there are many reports on the improvement of NAFLD by natural products, there are still numerous issues to be addressed regarding their actual efficacy and safety for consumption. These issues include adverse effects of berberine on the digestive system [15], the allergenicity of β-conglycinin [16], and the low efficacy of curcumin [17]. Therefore, continuously seeking safer and more effective natural bioactive substances is crucial for improving NAFLD.

Pea protein is a superior plant-based protein characterized by its balanced amino acid profile. Pea albumin (PA), which constitutes about 18–25% of pea protein, is abundant in sulfur-containing amino acids and other essential amino acids [17,18,19,20]. Research has found that PA is involved in lipid metabolism regulation. Liu et al. discovered that PA can regulate lipid metabolism in adipose tissue, alleviating obesity and related diseases [21]. Ruiz et al. demonstrated, through in vitro experiments, that PA hydrolysates significantly reduced lipid accumulation in 3T3-L1 cells [22]. Additionally, PA has been shown to inhibit colonic inflammation and modulate gut microbiota diversity. Miszkiewicz et al. found that PA possesses high free radical scavenging activity [23]. Dun et al. discovered that PA1F can regulate glucose metabolism in type 2 diabetic mice [24]. Therefore, PA might have the potential to alleviate hepatic lipid accumulation by regulating glucose and lipid metabolisms, oxidative stresses, and inflammatory responses associated with NAFLD development. Based on this analysis, we hypothesize that increasing the intake of PA in the diet could serve as an efficacious approach for the prevention and treatment of NAFLD. However, the effects and mechanisms of PA intervention in NAFLD are not yet clear.

In summary, we employed an HFD-induced NAFLD mouse model and administered oral PA during the induction period to evaluate the ameliorative effects of PA on hepatic steatosis in mice. The impacts of PA on NAFLD were assessed by analyzing changes in liver pathology, serum biochemical markers, and insulin resistance levels in the mice. Additionally, the expression of key proteins implicated in hepatic lipogenesis and lipolysis pathways was examined to explore the mechanisms by which PA influences the hepatic lipid metabolism. The findings from this study will contribute additional evidence to supporting the role of PA intervention in alleviating NAFLD.

## 2. Materials and Methods

### 2.1. Materials

Pea seed (*Pisum sativum* L.) was supplied by Yantai Shuangta Food Co., Ltd. (Yantai, China). Normal diet (ND, 10% energy from fat, D12450J) and high-fat diet (HFD, 60% energy from fat, D12492) products were obtained from SPF (Beijing, China) Biotechnology Co., Ltd. (Beijing, China). Triglyceride (TG, A110-1-1), total cholesterol (TC, A111-1-1), high-density lipoprotein cholesterol (HDL-C, A112-1-1), low-density lipoprotein cholesterol (LDL-C, A113-1-1), alanine aminotransferase (ALT, C009-2-1), aspartate aminotransferase (AST, C010-2-1), and superoxide dismutase (SOD, A001-3-2) assay kits were procured from Nanjing Jiancheng Bioengineering Institute (Nanjing, China). Malondialdehyde (MDA, S0131S) and glutathione peroxidase (GSH-Px, S0056) assay kits were purchased from Beyotime Biotech. Inc. (Nantong, China). The enzyme-linked immunosorbent assay (ELISA) kits for interleukin-1β (IL-1β, EK201B/3), interleukin-6 (IL-6, EK206/3), and tumor necrosis factor α (TNF-α, EK282/4) were purchased from Multisciences (Lianke) Biotech Co., Ltd. (Hangzhou, China). The ELISA kit for insulin (MM-0579M2) was obtained from Jiangsu Meimian Industrial Co., Ltd. (Yancheng, China). The primary rabbit antibodies against phospho-AMP-activated protein kinase alpha (*p*-AMPKα, AA393), AMPKα (AF1627), phospho-acetyl-CoA carboxylase (*p*-ACC, AA110), ACC(AF1867), sterol regulatory element binding transcription factor 1 (SREBF1, AF8055), fatty acid synthase (FASN, AG1915), peroxisome proliferator activated receptor-α (PPARα, AF7794), PPARγ (AF7797), and glyceraldehyde-3-phosphate dehydrogenase (GAPDH, AF1186) were obtained from Beyotime Biotech. Inc. (Nantong, China). The primary antibodies against adipose triglyceride lipase (ATGL, 55190-1-AP) was obtained from Proteintech Group, Inc. (Rosemont, IL, USA). The HRP-labeled goat anti-rabbit IgG(H + L) secondary antibody (A0208), ECL chemiluminescence kit (P0018FS) were purchased from Beyotime Biotech. Inc. (Nantong, China). The bicinchoninic acid (BCA) protein assay kit, Hematoxylin-eosin dye solution, and Oil Red O dye were procured from Beijing Solarbio Science & Technology Co., Ltd. (Beijing, China). All other chemicals and solvents were purchased from Sigma-Aldrich (St. Louis, MO, USA), unless otherwise described.

### 2.2. Preparation of Pea Albumin (PA)

The pea albumin (PA) was prepared following a previously described method, with minor adjustments [19]. In brief, ground pea seeds were treated to lipid extraction using hexane (1:3 *w*/*v*), and were subsequently air-dried. Next, the defatted meal was extracted (1:10 *w*/*v*) with 0.2 mol L^−1^ borate buffer (pH 8) containing 0.5 mol L^−1^ NaCl. The solution was centrifuged at 11,000× *g* for 30 min at 4 °C. The supernatant obtained was then adjusted to pH 4.5 using glacial acetic acid, stirred for 30 min at 4 °C, and subjected to centrifugation at 11,000× *g* for 30 min at 4 °C. The supernatant was dialyzed against distilled water and centrifuged at 11,000× *g* for 30 min at 4 °C. The supernatant was treated with 608 g L^−1^ (NH_4_)_2_SO_4_, stirred for 2 h at 4 °C, and centrifuged at 11,000× *g* for 30 min at 4 °C. The coacervate was collected and fully dissolved in water. After dialyzing against distilled water, the dialyzed extract (i.e., PA) was lyophilized and stored at −20 °C for further analysis. PA was characterized using SDS-PAGE to monitor the electrophoretic pattern. Specifically, a 15% separating gel and a 5% stacking gel were used to isolate PA. The electrophoresis conditions were set at 80 V for 40 min, followed by 120 V for 1 h. Finally, the gel was placed in R-250 staining solution for staining. After destaining, photographs were taken using a gel imaging system (ChemiScope 6100, Qinxiang, Shanghai, China). The protein concentration of PA was determined using the Kjeldahl method. The amino acid concentration in PA was analyzed using an amino acid analyzer (Biochrom 30+, BioChrom Ltd., Cambridge, UK).

### 2.3. Experimental Design and Animal Treatment

**Figure 1:**
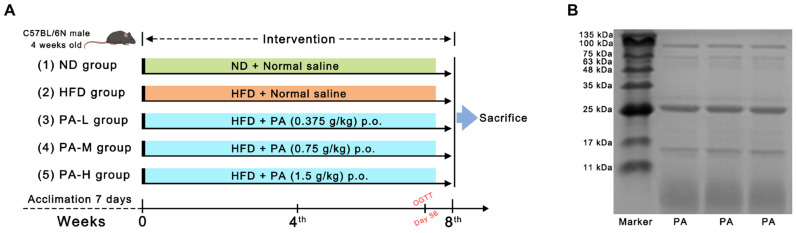
Experiment design and protein pattern of PA. (**A**) Detailed program of PA oral administration throughout the experiment period. (**B**) SDS-PAGE visualization for PA.

Forty male four-week-old C57BL/6N mice were obtained from Beijing Vital River Laboratory Animal Technology Co., Ltd. (Beijing, China). The mice were kept under standard conditions of ambient temperature (24 ± 2 °C) and relative humidity (50 ± 5%) with a 12 h light/dark cycle. After a one-week acclimatization period, mice were randomly divided into five groups (*n* = 8 per group): (1) normal diet (ND) group, fed with a normal diet and supplied with normal saline; (2) high-fat diet (HFD) group, fed with a high-fat diet and supplied with normal saline; (3) low-dose PA (PA-L) group, fed with a high-fat diet and supplied with 0.375 g/kg bw PA; (4) middle-dose PA (PA-M) group, fed with a high-fat diet and supplied with 0.75 g/kg bw PA; and the (5) high-dose PA (PA-H) group, fed with a high-fat diet and supplied with 1.5 g/kg bw PA. A detailed schedule of PA administration throughout the experiment period is shown in Figure 1A. The body weight of each group was recorded weekly over an 8-week trial. At the end of the 8-week intervention, the mice were sacrificed after an overnight fast. Blood samples were collected in test tubes. The serum was obtained using centrifugation (1000× *g*, 15 min, 4 °C) and stored at −80 °C for further study. Liver and white adipose tissue (including epididymal fat and subcutaneous fat) were collected and weighted. Parts of the liver tissues were fixed in 4% paraformaldehyde, while the remaining parts were quickly frozen in liquid nitrogen and stored at −80 °C until further processing. The experimental protocols were approved by the Institutional Animal Care and Use Committee of China Agricultural University (Approval Code: AW72303202-5-3).

### 2.4. Body Composition Measures

In the 8th week, the body fat composition of mice was measured using a nuclear magnetic resonance (NMR) analyzer (MesoQMR23-060H-I, NIUMAG, Suzhou, China). First, the device magnet temperature was stabilized to 32 °C, followed by instrument calibration. A standard oil sample was placed into the mouse restraint tube and positioned at the designated spot in the probe coil for self-testing. The peak signal coefficient between 0.99 and 1.01 indicated that the instrument was functioning normally. The NMR body composition analysis system was used to measure the fat mass of the mice, and the body fat content was expressed as fat mass/body weight × 100% [25,26]. Additionally, coronal images of the mice were obtained using the NMR imaging system. Initial parameter adjustments were performed, including automatic tuning of the center frequency, electronic shimming, and locating the soft pulse amplitude. After pre-scanning, image parameters and sequence parameters were set to complete the sampling of mice coronal images.

### 2.5. Oral Glucose Tolerance Test (OGTT) and Indexes of Insulin Resistance

An oral glucose tolerance test (OGTT) was conducted in the 7th week of the experimental timeline [21]. Following a 12 h fast, mice were given a glucose solution orally at a dosage of 2 g/kg body weight. Blood samples were drawn from the mice’s tail veins, and the glucose concentrations were determined at 0, 30, 60, 90, and 120 min after oral administration using the Yuwell 580 glucose meter (Yuwell Group, Danyang, China). After the test, the area under curve (AUC) for the blood glucose versus the time plot for each treatment group was calculated using integration. Following a 12 h fast at the conclusion of the 8th week, blood samples were taken from the tail vein to assess fasting blood glucose (FBG) concentration. Fasting insulin (FINS) was measured using an ELISA kit in accordance with the manufacturer’s guidelines. Insulin resistance was assessed using the Homeostasis Model Assessment-Insulin Resistance (HOMA-IR), which was calculated as HOMA-IR = FBG × FINS/22.5.

### 2.6. Histopathological Analysis

For the histological analyses, liver tissues were fixed in 4% paraformaldehyde for 24 h and were subsequently embedded in paraffin. Finally, liver tissues were then sectioned into 4 μm thick slices and stained using H&E [27]. The structure of liver tissue was observed under the Leica DM6B microscope (Leica, Wetzlar, Germany). The NAFLD activity score (NAS) was calculated by summing the individual scores for steatosis (0–3), hepatocellular ballooning (0–2), and lobular inflammation (0–3). In addition, liver tissues were embedded in an optimal cutting temperature (OCT) compound at −80 °C and were subsequently stained with Oil Red O. Stained liver tissue was observed with a Leica DM6B microscope (Leica, Wetzlar, Germany), and quantitative analysis was performed using ImageJ software (vision 1.8.0).

### 2.7. Serum and Hepatic Biochemical Analysis

Blood was collected from eyeballs under terminal anesthesia, and serum was collected by centrifuging at 1000× *g* at 4 °C for 15 min. The serum concentrations of TG, TC, HDL-C, and LDL-C, as well as the activities of ALT, and AST were analyzed according to the assay kits’ manufacturer’s instructions. Subsequently, the levels of TG and TC in the liver were measured with commercially available kits.

### 2.8. Liver Oxidative Stress and Inflammation Analysis

The concentrations of MDA, SOD, and GSH-Px in liver tissues were assayed to evaluate antioxidant capacity using commercially available kits. Subsequently, the protein levels of IL-1β, IL-6, and TNF-α in liver tissue were detected using an ELISA kit by following the manufacturer’s instructions.

### 2.9. Immunoblotting Analysis

Liver tissues were lysed using a RIPA buffer and were centrifuged to obtain the supernatant. The total protein concentrations were measured using a BCA protein assay kit. Protein samples with equivalent amounts were separated on 10% SDS-PAGE gels and were then transferred to PVDF membranes. The membranes were blocked using 5% BSA, followed by incubation with individual primary antibodies (*p*-AMPKα, AMPKα, *p*-ACC, ACC, SREBF1, FASN, PPARα, PPARγ, and ATGL) overnight at 4 °C. After washing, the membranes were then incubated with secondary antibodies at room temperature for 1 h. Following incubation, the PVDF membranes were washed. Finally, protein bands were visualized using an ECL kit. The protein expressions were quantified by using ImageJ vision 1.8.0 software with GAPDH for normalization.

### 2.10. Statistical Analysis

The analysis was conducted using the SPSS 23.0 software. Results were presented as mean ± SEM. The normality of the data were assessed using the Shapiro–Wilk test. Regarding the data with a normal distribution, statistical disparities between groups were analyzed with one-way analysis of variance (ANOVA) followed by an LSD post hoc test. For non-normally distributed data, the Kruskal–Wallis test was employed. *p* < 0.05 was considered to be statistically significant. Figures were created using GraphPad Prism 9.2.0.

## 3. Results

### 3.1. Characterization of Pea Albumin (PA)

The protein content of the PA prepared in this study was measured to be 92.65% using the Kjeldahl method. To determine the protein composition of PA, a SDS-PAGE analysis was conducted. The electrophoresis results are shown in Figure 1B, where four major bands can be clearly observed at 6, 16, 24, and 100 kDa. Among these, the protein bands with molecular weights of 6 kDa and 24 kDa accounted for the largest proportion. The four bands correspond to lipoxygenase (100 kDa), PA2 (24 kDa), trypsin inhibitor (16 kDa), and PA1 (6 kDa). Additionally, the amino acid composition of PA was analyzed, as presented in Table 1. The essential amino acids account for approximately 16.71%, glycine accounts for about 25.20%, and branched-chain amino acids (Leu, Val, Ile) account for about 7.34%.

### 3.2. PA Reduced HFD-Induced Weight Gain and Fat Accumulation in Mice

**Figure 2:**
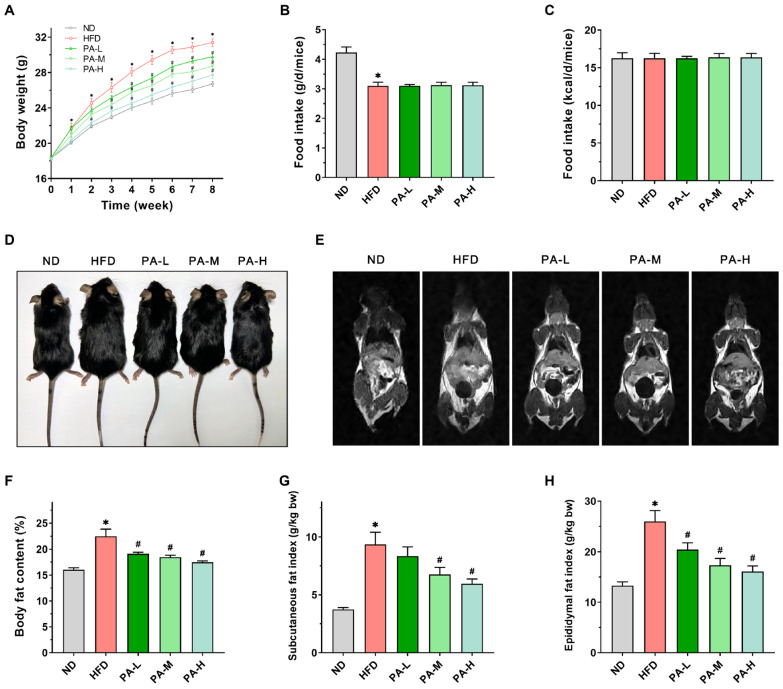
Effects of PA on body weight and fat accumulation in HFD-induced mice. (**A**) Body weight changes from week 0 to week 8. (**B**) Daily food intake per mouse in grams. (**C**) Daily energy intake per mouse. (**D**) Representative photos of the mice after 8 weeks. (**E**) Representative coronal sections of MRI studies. (**F**) Body fat percentage in mice. (**G**) Subcutaneous fat mass index. (**H**) Epididymal fat mass index. Results are expressed as mean ± SEM. *n* = 8 per group. * *p* < 0.05, HFD vs. ND; ^#^ *p* < 0.05, HFD vs. PA-L, PA-M, or PA-H.

HFD induction is a common method for establishing a mouse model of NAFLD. Given the frequent association between obesity and NAFLD in patients, we evaluated the body weight, food intake, and body fat content of the mice. During the 8-week experiment period, the mice were weighed weekly. The results indicate that, in comparison to the ND group, the HFD group exhibited a significant increase in body weight (Figure 2A, *p* < 0.05). By the eighth week, the body weight of the HFD group had risen by 17.5% compared to the ND group. Treatment with PA significantly inhibited the HFD-induced weight gain in a dose-dependent manner (*p* < 0.05). In terms of food intake, the mice fed a ND had significantly higher intake compared to those fed a HFD (Figure 2B, *p* < 0.05). Additionally, there was no difference in food intake between the HFD group and those treated with PA (Figure 2B, *p* > 0.05). However, no significant difference in energy intake was observed among all the groups (Figure 2C, *p* > 0.05). Through the appearance of the mice (Figure 2D) and MRI images (Figure 2E), it was evident that the HFD increased body fat in the mice, while the PA treatment group showed less fat accumulation. The body composition assessment indicated that feeding with a HFD significantly elevated the percentage of body fat mass in mice (Figure 2F, *p* < 0.05). In contrast, different doses of PA significantly reduced the percentage of body fat mass in a dose-dependent manner (*p* < 0.05). The relative weights of subcutaneous fat and epididymal fat can serve as references for the body fat ratio [28]. The subcutaneous fat and epididymal fat indices of the HFD group were significantly higher compared to the ND group (Figure 2G,H, *p* < 0.05). Nevertheless, PA treatment notably decreased the subcutaneous fat and epididymal fat index (*p* < 0.05) in a dose-dependent manner. The results indicated that PA intervention can reduce HFD-induced weight gain by inhibiting body fat accumulation.

### 3.3. PA Ameliorated Serum Lipid Profiles of HFD-Induced Mice

**Figure 3:**
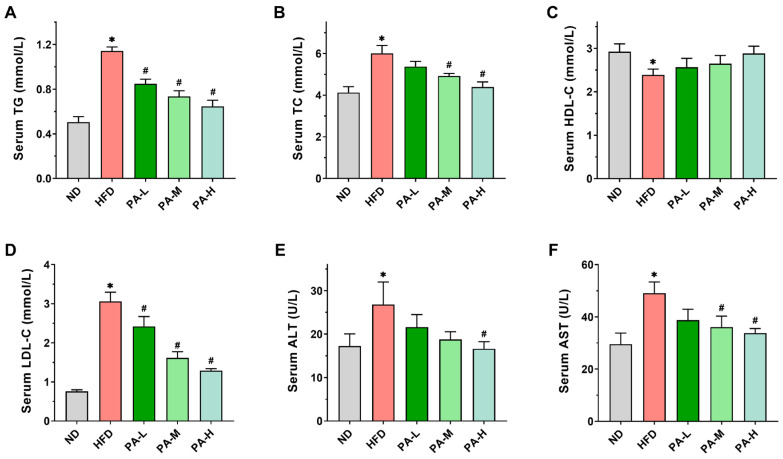
Effects of PA on serum lipid profiles in HFD-induced mice. (**A**–**D**) Serum TG, TC, HDL-C, and LDL-C levels. (**E**,**F**) Serum ALT and AST activities. Results are expressed as mean ± SEM. *n* = 8 per group. * *p* < 0.05, HFD vs. ND; ^#^ *p* < 0.05, HFD vs. PA-L, PA-M, or PA-H.

Disturbances in the lipid metabolism may result in lipid accumulation within the liver, promoting the progression of NAFLD [29]. To investigate the effects of PA on lipid metabolism in HFD-induced mice, we evaluated serum levels of TG, TC, HDL-C, and LDL-C, as well as the activities of AST and ALT. In the HFD group, serum TG levels were 2.26-fold higher compared to the ND group (Figure 3A), while serum TC levels showed a 1.45-fold increase relative to the ND group (Figure 3B). Additionally, serum LDL-C levels were 4.04 times higher in the HFD group than in the ND group (Figure 3D). However, PA intervention significantly reduced the serum levels of TG TC, and LDL-C in a concentration-dependent manner (*p* < 0.05). In the HFD group, serum HDL-C levels exhibited a significant reduction, decreasing by 18.33% compared to the ND group (Figure 3C, *p* < 0.05). After intervention with different doses of PA, the serum HDL-C levels increased by 7.41%, 10.85%, and 20.69% compared to the HFD group, respectively, although these changes were not statistically significant (*p* > 0.05). AST and ALT are typically used as biomarkers reflecting liver injury in NAFLD [30]. The serum ALT and AST activities in the HFD group were significantly elevated compared to those in the ND group (Figure 3E,F, *p* < 0.05). When orally administrated with PA, the serum ALT and AST activities reduced in a dose-dependent manner. The above data indicate that PA could effectively improve lipid metabolism abnormalities and liver injury induced by HFD, thereby alleviating the development of NAFLD.

### 3.4. PA Attenuated Hepatic Steatosis of HFD-Induced NAFLD Mice

**Figure 4:**
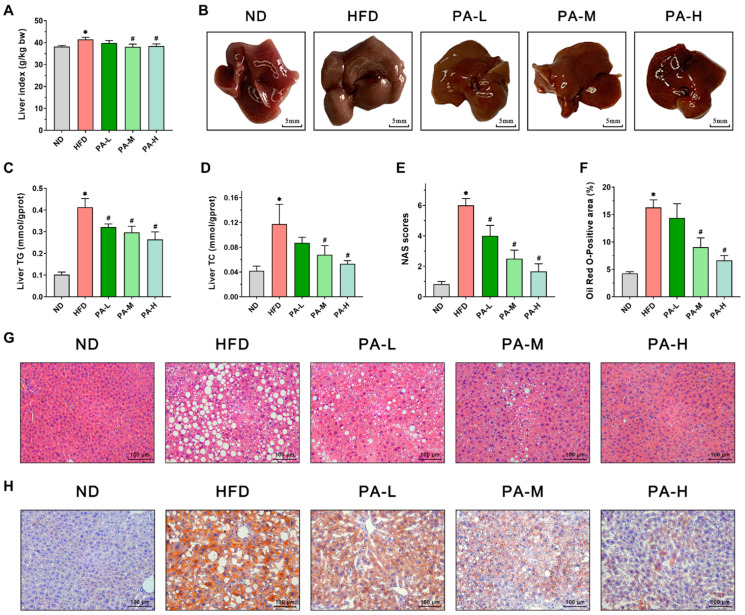
Effects of PA on hepatic steatosis in HFD-induced NAFLD mice. (**A**) Liver index. (**B**) Representative photos of livers. (**C**,**D**) Liver TG and TC levels. (**E**) NAFLD activity score of the liver. (**F**) Oil Red O-positive area of the liver. (**G**) Representative H&E stains of a liver section (Scale bar = 100 μm). (**H**) Representative Oil Red O stains of a liver section (Scale bar = 100 μm). Results are expressed as mean ± SEM. *n* = 8 per group. * *p* < 0.05, HFD vs. ND; ^#^ *p* < 0.05, HFD vs. PA-L, PA-M, or PA-H.

To assess the alleviating effects of PA on hepatic steatosis in NAFLD, we employed a HFD-induced NAFLD mice model. Liver weight, liver morphology, hepatic lipid profile, and liver tissue H&E and Oil Red O-stained sections were evaluated. As shown in Figure 4A, the liver index in the HFD group exhibited a significant 8.48% increase compared to the ND group (*p* < 0.05). However, PA intervention reduced the liver index, with the PA-M and PA-H groups showing significant reductions of 8.16% and 7.43%, respectively, compared to the HFD group (*p* < 0.05). Meanwhile, upon observing the liver morphology (Figure 4B), the livers of the ND group were reddish-brown with thin and smooth edges. In contrast, the livers of the HFD group mice appeared darker, with thickened and blunt edges, and were noticeably larger compared to the ND group. Following PA intervention, the liver color of the mice gradually returned from dark to a bright reddish-brown with increasing intervention doses, and the blunt edges diminished. Liver tissue lipid analysis showed that the TG and TC levels in the HFD group were significantly elevated compared to the ND group, increasing by 4.05 times and 2.79 times, respectively (Figure 4C,D, *p* < 0.05). PA intervention significantly reduced the liver levels of TG and TC in a concentration-dependent manner. These data indicate that PA could improve the hepatic lipid profile. Histopathological analysis of H&E stained liver sections showed that liver tissue structure in the ND group was normal, with no lesions (Figure 4G). In contrast, the HFD group exhibited steatosis, characterized by vacuolar degeneration, ballooning degeneration, and inflammatory infiltration. PA intervention effectively reduced the degree of hepatic steatosis, decreasing fat droplet accumulation, vacuolar degeneration, and inflammatory cell infiltration. Furthermore, high-dose PA intervention was the most effective in inhibiting hepatic steatosis. In the meantime, the NAS was significantly higher in the HFD group compared to the ND group (Figure 4E, *p* < 0.05). PA intervention resulted in a significant reduction in the NAS compared to the HFD group (*p* < 0.05). To further characterize hepatic steatosis, liver tissue was stained with Oil Red O. The results were similar to those observed with H&E staining. Significant lipid accumulation was observed in the liver of the HFD group, which markedly decreased with increasing doses of PA intervention (Figure 4H). Quantitative analysis of the Oil Red O-stained area in liver tissue indicated that the lipid droplet area in the HFD group was approximately 2.81 times larger than that in the ND group (Figure 4F, *p* < 0.05). After PA intervention, the lipid droplet area significantly decreased, reducing by approximately 11.73%, 44.43%, and 59.17% compared to the HFD group. In summary, the results indicated that PA intervention can significantly alleviate hepatic steatosis in NAFLD mice.

### 3.5. PA Inhibits Hepatic Lipid Accumulation by Modulating Lipid Synthesis and Degradation Pathways

**Figure 5:**
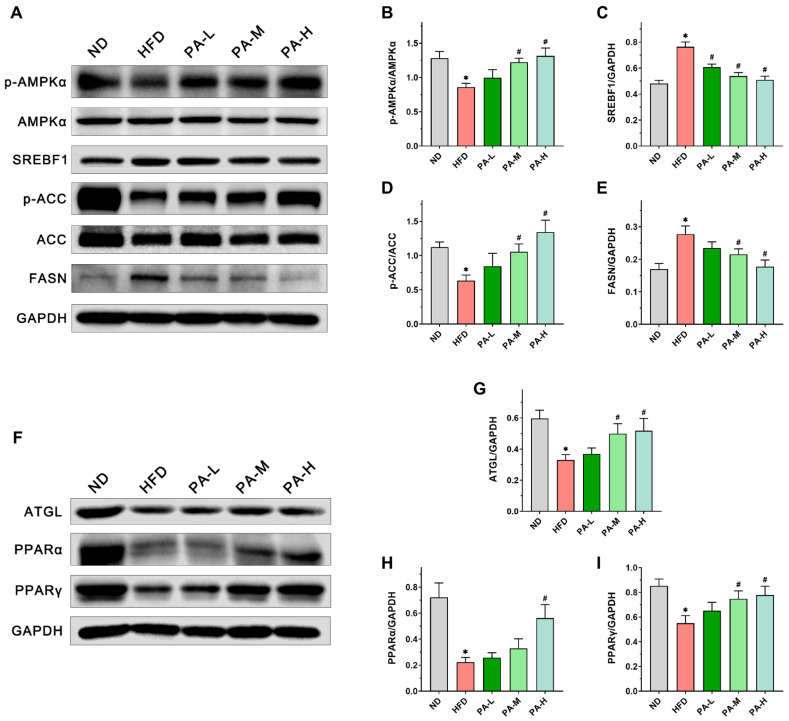
Effects of PA on the expression of lipid metabolism-related proteins in the liver of HFD-induced NAFLD mice. (**A**) Representative Western blots of markers for lipogenesis in the liver. (**B**–**E**) Quantification of protein expression of *p*-AMPKα/AMPKα, SREBF1, *p*-ACC/ACC, and FASN. (**F**) Representative Western blots of markers for lipolysis in the liver. (**G**–**I**) Quantification of protein expression of ATGL, PPARα, and PPARγ. Results are expressed as mean ± SEM. *n* = 6 per group. * *p* < 0.05, HFD vs. ND; ^#^ *p* < 0.05, HFD vs. PA-L, PA-M or PA-H.

To further assess the mechanisms by which PA alleviates NAFLD, we measured the expression of lipid metabolism-related proteins in the liver using immunoblotting. Firstly, the expression of AMPKα, SREBF1, ACC, and FASN, which are related to lipogenesis, has been evaluated. The findings indicated that PA treatment markedly increased the phosphorylation levels of AMPKα and ACC in a manner dependent on the dosage (Figure 5B,D, *p* < 0.05). Furthermore, PA was found to decrease the elevated expression of SREBF1 and FASN proteins in the livers of NAFLD mice induced with a HFD (Figure 5C,E, *p* < 0.05). In addition, we examined the effects of PA on the expression levels of lipolysis-related proteins such as ATGL, PPARα, and PPARγ. The intervention of PA dose-dependently prevented the decrease in ATGL, PPARα, and PPARγ protein expression induced with HFD (Figure 5G–I, *p* < 0.05). These data demonstrate that PA can alleviate NAFLD by inhibiting HFD-induced lipid synthesis and promoting lipid degradation.

### 3.6. PA Ameliorated Elevated Blood Glucose and Insulin Resistance of HFD-Induced Mice

**Figure 6:**
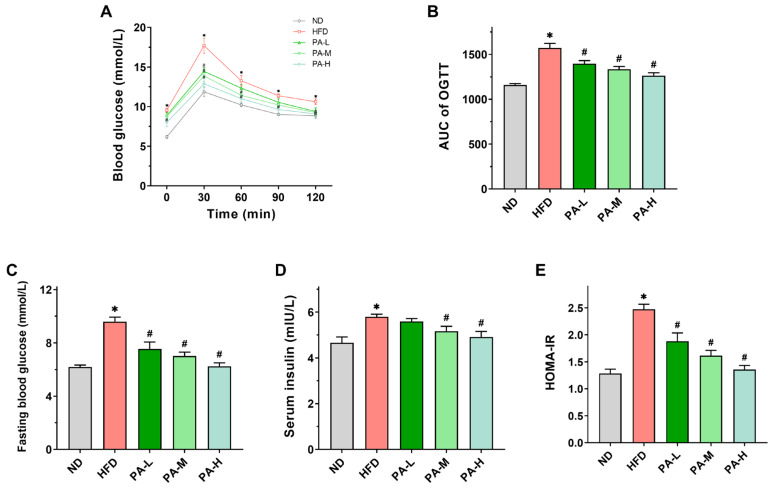
Effects of PA on glucose metabolism in HFD-induced mice. (**A**) Blood glucose profile of OGTT at week 7. (**B**) AUC measured during OGTT. (**C**) Fasting blood glucose levels in mice. (**D**) Serum insulin levels in mice. (**E**) HOMA-IR index in mice. Results are expressed as mean ± SEM. *n* = 8 per group. * *p* < 0.05, HFD vs. ND; ^#^ *p* < 0.05, HFD vs. PA-L, PA-M, or PA-H.

Insulin resistance constitutes a significant pathogenic mechanism in NAFLD [31], with OGTT and HOMA-IR serving as crucial indicators for its assessment [32]. Figure 6A illustrates that mice in the HFD group consistently showed markedly higher serum glucose levels across all time points. In contrast, mice receiving the PA intervention showed enhanced glucose tolerance at all time points during OGTT. The AUC value of the OGTT in the HFD group was approximately 1.36 times that of the ND group, while the high-dose PA intervention significantly reduced the AUC value by approximately 19.71% compared to the HFD group (Figure 6B, *p* < 0.05). These findings demonstrated that the PA intervention markedly enhanced glucose tolerance in mice induced with HFD. Additionally, fasting blood glucose and serum insulin concentrations were notably elevated in the HFD group compared to the ND group (Figure 6C,D, *p* < 0.05). The HOMA-IR value in the HFD group was notably higher than that in the ND group (Figure 6E, *p* < 0.05). The PA intervention significantly mitigated the HFD-induced elevations in fasting blood glucose levels, serum insulin concentrations, and HOMA-IR values in a dose-dependent manner. The results indicated that PA intervention could significantly improve HFD-induced insulin resistance.

### 3.7. PA Attenuates Hepatic Oxidative Stress and Inflammatory Response of HFD-Induced Mice

**Figure 7:**
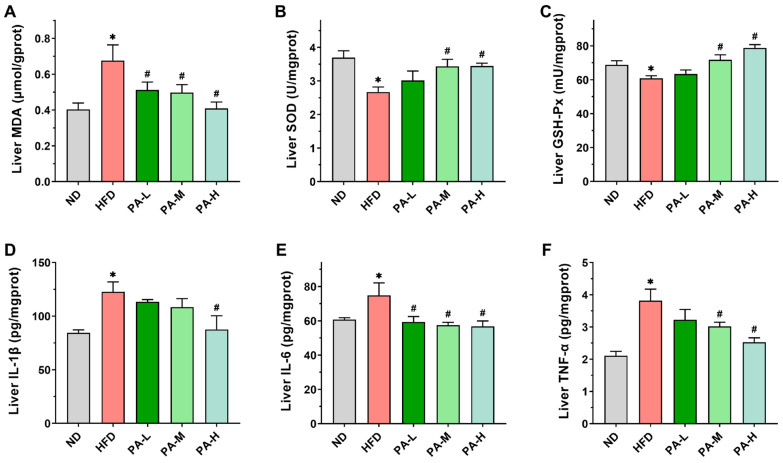
Effects of PA on hepatic oxidative stress and inflammatory response in HFD-induced mice. (**A**–**F**) Levels of MDA, SOD, GSH-Px, IL-1β, IL-6, and TNF-α in the liver of mice. Results are expressed as mean ± SEM. *n* = 8 per group. * *p* < 0.05, HFD vs. ND; ^#^ *p* < 0.05, HFD vs. PA-L, PA-M, or PA-H.

Various studies have shown that the development of NAFLD is always accompanied by oxidative stress and inflammatory responses [33,34,35]. To evaluate hepatic oxidative stress, levels of MDA and activities of SOD and GSH-Px in the liver were measured. In comparison to the ND group, mice in the HFD group exhibited markedly elevated MDA levels (Figure 7A, *p* < 0.05) and significantly decreased activities of SOD and GSH-Px in the liver (Figure 7B,C, *p* < 0.05). The PA intervention markedly decreased hepatic MDA levels and substantially enhanced SOD and GSH-Px activities in a dose-dependent manner (*p* < 0.05), confirming the antioxidant effects of PA in the liver. Next, we evaluated the effect of PA on the hepatic levels of pro-inflammatory cytokines IL-1β, IL-6, and TNF-α. The concentrations of hepatic IL-1β, IL-6, and TNF-α were significantly elevated in the HFD group compared to the ND group (Figure 7D–F, *p* < 0.05). However, high-dose PA intervention significantly reduced the hepatic levels of IL-1β, IL-6, and TNF-α (*p* < 0.05). These findings suggest that PA can effectively suppress HFD-induced hepatic inflammation.

## 4. Discussion

NAFLD, the most prevalent metabolic liver disease globally, is characterized by hepatic steatosis [36]. Its pathogenesis is closely associated with insulin resistance, fatty acid synthesis, lipid peroxidation, oxidative stress, and gut microbiota dysbiosis. Currently, no specific drugs are available worldwide for the prevention or alleviation of NAFLD. The search for natural active substances to alleviate NAFLD is a potential intervention and treatment strategy. In our earlier studies, we showed that PA has beneficial effects in alleviating HFD-induced obesity [21]. Therefore, increasing the intake of PA in the diet could be an effective strategy for preventing and treating NAFLD. In this study, we determined the protein profile of PA using SDS-PAGE (Figure 1B). PA mainly consists of four proteins: lipoxygenase (100 kDa), PA2 (24 kDa), trypsin inhibitor (16 kDa), and PA1 (6 kDa), which are consistent with the composition reported in the literature [37]. It has been found that amino acid composition is related to the development of NAFLD. Glycine can alleviate NAFLD by promoting fatty acid β-oxidation and glutathione synthesis, while increased intake of branched-chain amino acids can significantly improve liver function [38]. Amino acid composition analysis of PA revealed that glycine accounts for approximately 25.20% and branched-chain amino acids account for approximately 7.34% of PA (Table 1). These results suggest that PA possesses material basis to alleviate NAFLD.

HFD induction is a common method for establishing an NAFLD mouse model. To evaluate the ameliorative effects of PA on NAFLD, we established an NAFLD mouse model induced with a HFD for 8 weeks. Mice in the HFD group exhibited significantly increased body weight, body fat percentage, serum lipid levels, serum transaminase levels, and insulin resistance. Additionally, lipid accumulation, steatosis, oxidative stress, and inflammatory damage were observed in the liver. According to the NAS assessment based on the criteria proposed by the NASH Clinical Research Network [39], the liver tissues of mice induced with a HFD were pathologically evaluated. In this study, the NAS of the HFD-induced mice reached 6, which is a diagnostic for NASH when the score exceeds 5. These comprehensive results indicated the successful establishment of the HFD-induced NAFLD mouse model, which is consistent with previously reported findings in the literature [40,41].

To investigate the effects and mechanisms of PA in alleviating NAFLD, we administered different doses of PA to the mice via gavage simultaneously with the induction of the NAFLD model. We found that PA can alleviate HFD-induced hepatic lipid accumulation by modulating serum lipid profiles, reducing insulin resistance, and enhancing antioxidant and anti-inflammatory capacities. The underlying mechanisms are likely mediated through the regulation of key proteins involved in hepatic lipogenesis, lipolysis, and fatty acid oxidation pathways. Currently, the most widely accepted pathogenesis mechanism of NAFLD is the “two-hit” hypothesis. The initial hit involves the accumulation of lipids, primarily triglycerides, in hepatocytes, leading to steatosis [4,42]. This study found that PA intervention substantially alleviated hepatic steatosis caused by the HFD. Specifically, morphological and histological examinations revealed that PA significantly reduced fat deposition in the liver. Compared to the HFD group, low, medium, and high doses of PA intervention reduced the area of red lipid droplets in Oil Red O staining of mice liver by 11.73%, 44.43%, and 59.17%, respectively. Additionally, the levels of TG and TC in the livers of PA-treated mice were markedly lower compared to those in the HFD group. These results suggest that PA significantly reduced hepatic lipid accumulation. Prolonged lipid retention in the liver frequently induces liver damage, potentially worsening NAFLD progression [5]. H&E staining and NAS results showed that PA intervention significantly reduced the number of hepatic fat vacuoles, ballooning degeneration, and inflammatory cell infiltration compared to the HFD group, with a significant decrease in NASs. While H&E staining and NAS are standard methods for pathological assessment, they depend on the subjective judgment of pathologists, potentially leading to inconsistencies in scoring. Consequently, we quantified the levels of commonly used biomarkers of liver function, specifically serum ALT and AST. When hepatocytes are in a pathological state of damage, they release ALT and AST into the bloodstream [43]. Therefore, increased levels of ALT and AST in the serum indicate a certain degree of liver damage. In this experiment, it was observed that PA intervention significantly reduced serum ALT and AST activities compared to the HFD group. The results demonstrated that PA mitigated HFD-induced liver damage. However, elevated levels of ALT and AST are typically observed when liver damage is already severe, making these biomarkers potentially unsuitable for the early detection or prediction of liver disease. Therefore, we also evaluated the effects of PA on the serum lipid profile, insulin resistance, oxidative stress, and inflammation levels to elucidate how PA intervention reduces hepatic steatosis and liver damage. The liver disease induced with a HFD in mice closely resembles the pathogenesis of human NAFLD. Ipsen et al. demonstrated that cereal proteins can improve lipid metabolism disorders and alleviate liver damage [44]. Lemus-Conejo et al. showed that lupin peptides can reduce AST and ALT related to liver damage, thereby alleviating HFD-induced NAFLD in mice [45]. Our study results also indicated that PA intervention can reduce HFD-induced hepatic steatosis and liver damage. Therefore, increasing PA intake as a dietary supplement might be an effective strategy for preventing and treating human NAFLD.

Disorders in the lipid metabolism can exacerbate the accumulation of fat in hepatocytes, contributing to the development and advancement of NAFLD and subsequently impairing liver function [46]. This study found that PA significantly enhanced the serum lipid profile by lowering serum levels of TG, TC, and LDL-C, while increasing the levels of HDL-C in mice. These findings align with prior research indicating that supplementation with pea protein can reduce serum cholesterol levels by modulating the microbiome [47]. Naik et al. found that cereal proteins can increase serum HDL concentrations [48]. Studies have shown that the phosphorylation of AMPK induces the phosphorylation of ACC, thereby reducing ACC activity and inhibiting TG formation. Therefore, PA may improve the serum lipid profile in mice by activating the AMPK pathway [49]. Given the critical role of lipid metabolism disorders in NAFLD pathogenesis, the improvement in serum lipid profile by PA intervention may have beneficial effects in alleviating NAFLD. Additionally, insulin resistance represents the predominant metabolic anomaly in NAFLD, with established research highlighting its close association with the initial pathogenic mechanisms of NAFLD [50,51]. The activation of the hepatic de novo lipogenesis pathway induced by insulin resistance contributes significantly to lipid accumulation in NAFLD [52]. Insulin resistance decreases the liver’s ability to convert glucose into glycogen, leading to excessive synthesis of fatty acids from the surplus glucose, which are then esterified into TG, resulting in the excessive accumulation of TG in hepatocytes [53]. Research has shown that in NAFLD patients, metabolic disorders such as insulin resistance lead to a liver fat synthesis rate that is three times higher than in normal individuals [54]. Therefore, intervening in insulin resistance is an important approach to alleviate hepatic lipid accumulation. Ofosu et al.’s research indicates that grain bioactive peptides can alleviate metabolic syndrome, including type 2 diabetes and obesity [55]. This experiment found that PA could improve glucose tolerance, lower fasting blood glucose levels, and alleviate insulin resistance in NAFLD mice in a dose-dependent manner. Studies have shown that HFD feeding leads to increased phosphorylation of insulin receptor substrate 1 (IRS1), therefore inhibiting the IR/IRS-1 interaction and ultimately causing insulin resistance [56]. Liu et al. demonstrated that PA can inhibit IRS1 phosphorylation [21]. Therefore, in this study, PA may alleviate a HFD-induced insulin resistance by inhibiting the IRS1 phosphorylation pathway. These findings suggest that PA intervention can alleviate hepatic lipid accumulation in NAFLD by reducing insulin resistance and regulating lipid metabolism disorders induced with a HFD.

The “second hit” in NAFLD involves hepatic oxidative stress caused by excessive lipid accumulation, resulting in the production of significant ROS quantities [57]. When liver tissue is exposed to ROS, it triggers an inflammatory response, thereby accelerating the progression towards NASH [4]. Therefore, inhibiting oxidative stress and inflammatory responses represents a pivotal strategy for intervening in NAFLD. Under normal physiological conditions, cells can eliminate ROS and uphold cellular balance via internal antioxidant defense mechanisms like SOD and GSH-Px [58]. However, under oxidative stress in the liver, mitochondria become damaged, leading to the reduced metabolism of SOD and GSH-Px and an increase in MDA levels [59]. The findings from this research revealed a significant increase in liver MDA levels and a notable decrease in SOD and GSH-Px activities in the HFD group, indicating that HFD induced hepatic oxidative stress. In contrast, PA intervention significantly enhanced the activities of SOD and GSH-Px in the liver and reduced the MDA content, suggesting that PA intervention can alleviate HFD-induced oxidative stress in the liver. Research has shown that regulating the Nrf2 signaling pathway can modulate antioxidant enzymes such as NQO1, HO-1, providing protective effects against oxidative stress-induced cellular damage [60]. Specific upregulation of Nrf2 has been used to treat NAFLD induced by long-term HFD [61]. Ran et al. discovered that activation of the Nrf2/HO-1 signaling axis in the liver of HFD-fed mice can maintain antioxidant levels [33]. Zhu et al. found that activating the Nrf2/ARE pathway exerts antioxidant effects, thus preventing and treating NAFLD [40]. Therefore, PA may inhibit HFD-induced hepatic oxidative stress by modulating the Nrf2 signaling pathway. Studies have established that the development of NAFLD is also regulated by pro-inflammatory cytokines IL-1β, IL-6, and TNF-α [62]. Our study revealed significant elevation of IL-1β, IL-6, and TNF-α levels in the livers of HFD-induced NAFLD mice, whereas PA intervention markedly decreased these cytokine levels. Similarly, previous research has also found that PA can effectively inhibit the elevation of IL-1β, IL-6, and TNF-α levels in colitis [20]. The research confirmed that activation of TLR4 leads to the activation of NF-κB, promoting the release of pro-inflammatory cytokines and triggering liver inflammation [63]. Zhao et al. demonstrated that inhibiting the TLR4/NF-κB/NLRP3 signaling pathway can regulate the secretion of inflammatory factors to improve NAFLD [64]. Wang et al. found that the TLR4-NF-κB pathway is a key mechanism in mitigating inflammation damage in NAFLD [34]. Our earlier research also found that PA has the ability to block the NF-κB and STAT3 signaling pathways [20]. Based on these findings, we believe that the improvement of inflammatory response in NAFLD by PA is associated with the inhibition of the hepatic TLR-4/NF-κB pathway. The above results demonstrate that PA intervention can mitigate the “second hit” of HFD-induced NAFLD by suppressing oxidative stress and inflammatory responses.

Based on the above conclusions, we confirm that PA can alleviate HFD-induced NAFLD. Next, we investigated the mechanisms through which PA regulates hepatic lipid accumulation in NAFLD. Hepatic lipid metabolism primarily involves de novo lipogenesis, triglyceride synthesis, triglyceride hydrolysis, and fatty acid oxidation [65]. These hepatic lipid metabolism processes are regulated by various proteins. FASN and ACC serve as rate-limiting enzymes in fatty acid synthesis [66], while SREBF1 is a key transcription factor that regulates fatty acid synthesis by controlling the expression of crucial enzymes like FASN and ACC [67]. As a key energy sensor and regulator of the systemic metabolism, AMPK plays a vital role in maintaining lipid metabolism balance [68]. Phosphorylation of AMPK can inhibit the expression of SREBF1 while increasing the phosphorylation level of ACC, promoting the inactivation of ACC [69,70]. Lee et al. demonstrated that activating AMPK phosphorylation can enhance ACC phosphorylation, thereby inhibiting the expression of SREBF1 and FASN, reducing hepatic lipid accumulation [71]. Kawaguchi et al. indicated that wheat bran peptides can alleviate HFD-induced NAFLD in mice by upregulating the AMPK/ACC pathway [72]. ATGL serves as the rate-limiting enzyme in triglyceride hydrolysis, primarily functioning to convert triglycerides into free fatty acids and glycerol [73]. Research has shown that PPARα is crucial in the hepatic lipid metabolism by enhancing the transcription levels of various fatty acid oxidation proteins, thereby promoting mitochondrial fatty acid oxidation [74]. Additionally, ATGL deficiency can reduce the expression of the PPARα gene, leading to impaired mitochondrial fatty acid oxidation function [75]. Research has shown that the activation of PPARγ can reduce the transfer of fatty acids to the liver [76], decrease insulin resistance [77], and inhibit the expression of IL-6 and TNF-α [78]. In this research, HFD induction led to an upregulation of SREBF1 and FASN expression in the liver, a decrease in the phosphorylation of AMPKα and ACC, and a downregulation of ATGL, PPARα, and PPARγ expression. On the contrary, PA intervention can increase the phosphorylation of AMPKα and ACC, reduce the expression of SREBF1 and FASN, and enhance the expression of ATGL, PPARα, and PPARγ, thereby inhibiting de novo lipogenesis while promoting triglyceride hydrolysis and mitochondrial fatty acid oxidation. From these results, it can be concluded that PA alleviates hepatic steatosis by modulating the lipid metabolism, thereby mitigating the progression of NAFLD.

## 5. Conclusions

**Figure 8:**
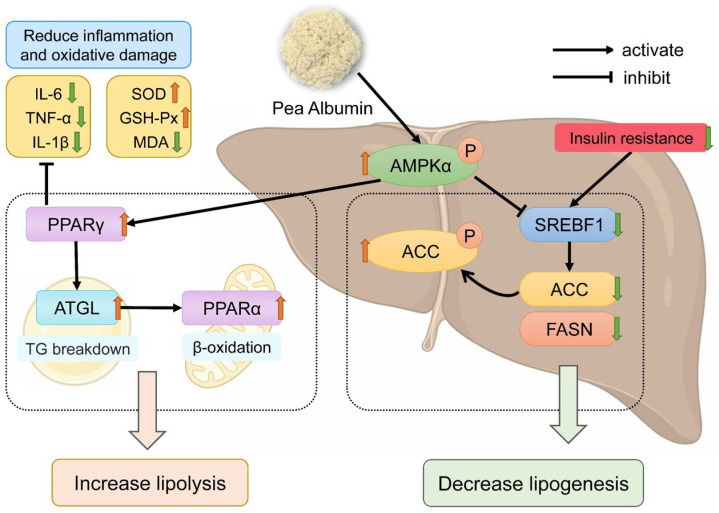
Schematic illustration of the mechanism of pea albumin in ameliorating HFD-induced NAFLD. The red upward arrows represent increase; the green downward arrows represent decrease.

In conclusion, PA intervention can improve dyslipidemia, insulin resistance, oxidative stress, and inflammatory responses induced with a high-fat diet intake, thereby reducing hepatic steatosis, and alleviating the development of NAFLD. PA reduces hepatic lipid accumulation in mice by regulating the expression of proteins involved in hepatic lipogenesis and lipolysis (Figure 8). However, this study has some limitations, such as not determining the exact manner in which PA is absorbed in the intestines or the form in which PA is transported through the blood to ultimately target the liver. Therefore, further research is needed to isolate PA to identify the specific components within PA that regulate lipogenesis and lipolysis, in order to better understand its potential mechanisms of action. Overall, PA can serve as a natural dietary supplement to provide a new strategy for improving NAFLD.

## Figures and Tables

**Table 1:** Protein content and amino acid composition of PA.

Items	Amount
Protein (%)	92.65 ± 0.66
Essential amino acids (%)	
Lysine	3.92 ± 0.01 ^h^
Leucine	3.81 ± 0.02 ^i^
Threonine	2.52 ± 0.01 ^j^
Valine	2.26 ± 0.03 ^k^
Phenylalanine	1.91 ± 0.01 ^l^
Isoleucine	1.27 ± 0.02 ^m^
Tryptophan	0.73 ± 0.02 ^o^
Methionine	0.29 ± 0.02 ^p^
Nonessential amino acids (%)	
Glycine	25.20 ± 0.05 ^a^
Proline	15.89 ± 0.02 ^b^
Alanine	11.14 ± 0.04 ^c^
Glutamate	10.20 ± 0.02 ^d^
Arginine	8.47 ± 0.01 ^e^
Aspartate	6.09 ± 0.03 ^f^
Serine	4.31 ± 0.01 ^g^
Tyrosine	0.99 ± 0.01 ^n^
Histidine	0.99 ± 0.04 ^n^

Values are expressed as mean ± SD (*n* = 3). Different superscript letters indicate significant differences (*p* < 0.05) among the amino acid content.

## Data Availability

The data presented in this study are available on request from the corresponding author. The data are not publicly available due to privacy and ethical reasons.
